# Purification and Preliminary Crystallographic Analysis of a New Lys49-PLA2 from *B. Jararacussu*

**DOI:** 10.3390/ijms9050736

**Published:** 2008-05-08

**Authors:** Marcelo L. dos Santos, Fábio H. R. Fagundes, Bruno R. F. Teixeira, Marcos H. Toyama, Ricardo Aparicio

**Affiliations:** 1 Laboratório de Biologia Estrutural e Cristalografia, Instituto de Química, Universidade Estadual de Campinas, CP 6154, 13083–970, Campinas-SP, Brazil; 2 Instituto de Biologia, Universidade Estadual de Campinas, Campinas-SP, Brazil; 3 Laboratório de Química de Macromoléculas, UNESP/CLP, São Vicente-SP, Brazil

**Keywords:** Lys49-PLA2, *Bothrops jararacussu*, platelet aggregation, phospholipase crystallographic analysis

## Abstract

BjVIII is a new myotoxic Lys49-PLA2 isolated from *Bothrops jararacussu* venom that exhibits atypical effects on human platelet aggregation. To better understand the mode of action of BjVIII, crystallographic studies were initiated. Two crystal forms were obtained, both containing two molecules in the asymmetric unit (ASU). Synchrotron radiation diffraction data were collected to 2.0 Å resolution and 1.9 Å resolution for crystals belonging to the space group *P*2_1_2_1_2_1_ (*a* = 48.4 Å, *b* = 65.3 Å, *c* = 84.3 Å) and space group *P*3_1_21 (*a* = *b* = 55.7 Å, *c* = 127.9 Å), respectively. Refinement is currently in progress and the refined structures are expected to shed light on the unusual platelet aggregation activity observed for BjVIII.

## 1. Introduction

Phospholipases A2 (PLA2; EC 3.1.1.4) are a family of enzymes that catalyze the hydrolysis of the *sn*-2 ester bond of phospholipids to release free fatty acids, including arachidonic acid [1]. In addition to its enzymatic function, snake venom PLA2 can be neurotoxic, myotoxic and cardiotoxic [2]. Many of these properties are conferred by regions of the structure not involved in catalysis, as illustrated by the myotoxicity of the minimally catalytically active subgroup of PLA2 homologues, that possess a lysine at position 49 in the amino acid sequence (Lys49-PLA2) [3, 4].

Snake venom components can also affect the homeostatic system by the inhibition or potentiation of some physiological events associated with blood coagulation. These components usually belong to various families such as serine proteases, metalloproteinases, C-type lectins, disintegrins and phospholipases A2 [5]. At the present time, PLA2s can be classified into three different groups according to their effect on blood coagulation. The first group includes PLA2s that induce platelet aggregation; the second group acts as physiological agonists of platelet aggregation and the third group is characterized by a biphasic response on platelet aggregation (pro- and anti-aggregation properties) [6]. In the case of PLA2s isolated from the Bothrops genus, only enzymatically active phospholipases with an aspartic acid at position 49 (Asp49-PLA2), such as BhtX-II, have been characterized as potent activators of platelet reactions [7]. Its platelet aggregation activity involves cellular signaling activation, including protein kinase C, adenylyl cyclase activation pathways and also thromboxane A2 formation in the primary reaction [7]. Several enzymatically active PLA2s have been characterized as strong anticoagulant compounds as previously described by Magro *et al.* [8] and Higuchi *et al.* [2]. From the primary structure point of view, all these proteins are enzymatically active Asp49-PLA2s.

Here, we present a new pro-platelet aggregation non-catalytically active Lys49-PLA2 isolated from the *Bothrops jararacussu* venom, which will be referred to as BjVIII. Interestingly, similarly to other Lys49-PLA2s, BjVIII does not present significant enzymatic activity but, at the same time, it is able to induce a strong platelet aggregation typical of enzymatically active Asp49-PLA2s. This unusual behavior suggests the existence of regions in the protein structure, distinct from the active site, related to platelet aggregation activity.

This work describes the isolation of BjVIII from *B. jararacussu* venom and its characterization with respect to its unexpected platelet agreggation activity. In addition, a preliminary X-ray diffraction analysis of BjVIII in two different crystal forms is presented. The refined structures are expected to shed light on the unusual properties observed for this new Lys49-PLA2.

## 2. Materials and methods

### 2.1. Protein purification

BjVIII was purified from *Bothrops jararacussu* whole venom by a two-step chromatographic procedure according to the methods described by Toyama *et al.* [9] and Fonseca *et al.* [10]. Initially, 10 mg of the crude venom was dissolved in 250 μL loading buffer (0.05 M Tris-HCl, pH 8.0) and centrifuged at 4500 × g for 5 minutes. The supernatant was injected into a BioSuite Q AXC ion exchange column (Waters). Fractions were eluted with a gradient of a buffer containing 0.05 M Tris-HCl, pH 8.0, with increasing concentrations of 1.0 M NaCl (0–100%) at a constant flow rate of 1 mL min^−1^. All fractions were collected and individually analyzed for myotoxicity, phospholipasic A2 activity and by tricine SDS-PAGE following the method described by Schägger & von Jagow [11]. The BjVIII fractions obtained in the first chromatographic step were dissolved in 250 μL of an aqueous solution containing 0.15% trifluoroacetic acid. The supernatants were injected into a X-Terra C18 analytical reverse phase column, followed by elution with a mobile phase of 0.15% aqueous trifluoroacetic acid with increasing quantities of 66% acetonitrile (0–100%). The degree of purity of BjVIII was assessed by SDS-PAGE.

### 2.2. Platelet aggregation assays

Human venous blood was collected with informed consent from healthy volunteers who denied taking any medication in the previous 14 days. Blood was collected by a two-syringe technique using polypropylene syringes with 19-gauge needles, and immediately transferred into polypropylene tubes previously containing 1/10th of the tube final volume of 3.8% trisodium citrate. Initially, whole blood was centrifuged to obtain the platelet-rich plasma (PRP) and, after removing the PRP, the remaining blood was centrifuged at 3000 × g for 5 minutes to obtain washed platelet. The platelet aggregation assays were conducted with a washed platelet preparation that was left for 1 h at room temperature to recover its sensitivity to aggregation agents. Platelet counts were performed on a Coulter S Plus (Coulter Electronics) and by phase-contrast microscopy. Platelet aggregation was measured turbidimetrically using a dual-channel whole blood Lumi-aggregometer (Chrono Log Corporation). Platelets suspended in a phosphate buffered saline buffer (400 μL) were pre-incubated at 37°C for 2 minutes under stirring with 1 mM CaCl_2_ (final concentration) and challenged with BjVIII or other proteins in the presence or absence of inhibitors. The aggregation was recorded after 7 minutes from the application of the toxins.

### 2.3. Crystallization experiments

The lyophilized sample of native BjVIII was dissolved in ultra-pure water at a concentration of 10 mg mL^−1^. Crystallization conditions were initially screened by the hanging-drop vapour-diffusion method [12] at 20°C using the Crystallization Basic and Extension Kits for Proteins (Sigma-Aldrich). Crystallization drops were prepared by mixing 2 μL of protein solution and an equal volume of the precipitant solution, and equilibrated against 500 μL of the same precipitant solution using 24-well tissue culture test plates (TPP). Small crystals were found in condition number 40 of the Crystallization Basic Kit (0.1 M sodium citrate, pH 5.6, 20% *v/v* 2-propanol and 20% *w/v* PEG 4000) and in number 26 of the Crystallization Extension Kit (0.2 M ammonium sulfate, 0.1 M MES, pH 6.5 and 30% *w/v* PEG MME 5000). These initial crystallization conditions were refined (section *Results and discussion*) and better crystals, suitable for data collection, were obtained.

### 2.4. X-ray data collection and processing

Crystals were cryoprotected using reservoir solution supplemented with 20% *v/v* ethylene glycol and rapidly frozen in a nitrogen-gas stream (Oxford Cryosystems). X-ray diffraction data were collected at the wavelength of 1.425 Å at the Laboratório Nacional de Luz Síncrotron (LNLS), Campinas, Brazil, beamline D03B-MX1 [13, 14], using a MAR CCD 165 detector (MAR Research). Diffraction data were integrated with *MOSFLM* [15] and scaled using *SCALA* [16]. Structures of BjVIII in both crystal forms were solved by the Molecular Replacement method using the program package *AMoRe* [17], calculations were carried out using a resolution range of 15.0−4.0 Å and default parameters. Structural superpositions were done with *SUPCOMB* [18]. Further analyses were performed using programs from the *CCP4* suite [19, 20].

## 3. Results and discussion

### 3.1. Purification of Lys49-PLA2 BjVIII

**Figure 1.**
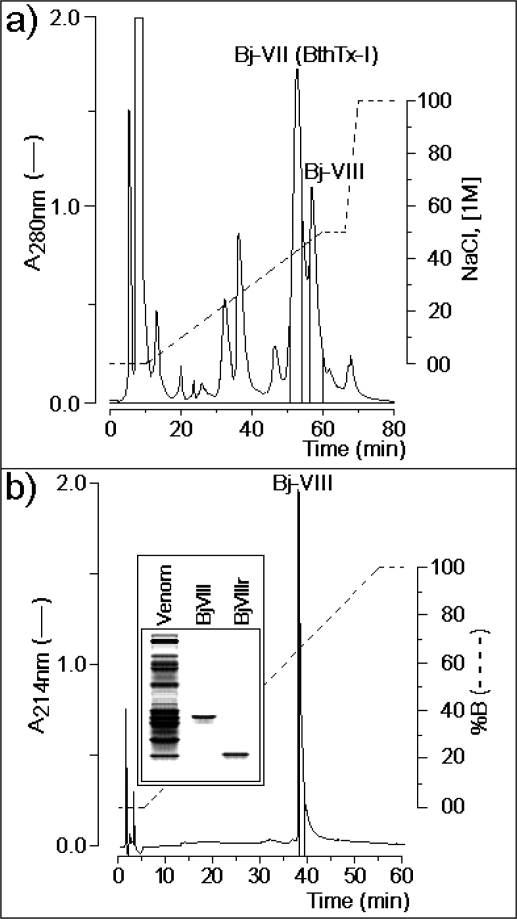
a) Ion exchange chromatograph of *Bothrops jaracussu* venom, where Bj-VII (BthTx-I) represents the main myotoxic Lys49-PLA2 and BjVIII is a novel PLA2. b) Reverse phase chromatograph of BjVIII PLA2. The insert shows the tricine SDS-PAGE electrophoresis of native BjVIII and BjVIII treated with 1 M DTT (BjVIIIr). The dashed line in a) represents the gradient of buffer containing 0.05 M Tris-HCl, pH 8.0, to which 1.0 M NaCl is added, while the dashed line in b) shows the increasing concentrations of 66% acetonitrile added to aqueous 0.15% trifluoroacetic acid (solution B).

Bothrops toxin I (BthTx-I) was the only Lys49-PLA2 purified from *Bothrops jaracussu* venom by one chromatographic step [21]. We modified the original protocol, using a two-step chromatographic procedure (section *Materials and methods*) and observed that BthTx-I fraction from the one-step protocol is composed of two closely related isoforms. This new BthTx-I-like isoform was referred to as BjVIII. Chromatographic integration indicates that, BthTx-I and BjVIII account for approximately 26% and 11% of whole venom, respectively (Figure 1).

The BjVIII fraction was collected and an aliquot of this fraction was submitted to a treatment with 1 M DTT; this sample is referred to as BjVIIIr. Native BjVIII and BjVIIIr were analyzed by polyacrylamide gel electrophoresis under non-reducing conditions and revealed the presence of a single protein band in both cases (Figure 1b). Native BjVIII showed a molar mass of approximately 29 kDa, while the protein subjected to the treatment with DTT showed a molar mass of approximately 14 kDa, corresponding to the dimeric and monomeric BjVIII forms, respectively. The same behavior, under similar conditions, was observed for another Lys49-PLA2 isolated from *Bothrops neuwiedi pauloensis* venom [22]. These results indicate that BjVIII probably assumes a dimer-like structure in solution, as verified for some other Lys49-PLA2s homologues by electrophoretic, spectroscopic and small angle X-ray scattering (SAXS) studies [23, 24].

### 3.2. Atypical platelet aggregation activity of BjVIII

A detailed biochemical characterization of BjVIII will be published elsewhere (Fagundes *et al.*, to be published). Similarly to other Lys49-PLA2s, such as PrTx-I, PrTx-II [25] and BthTx-I [21], BjVIII does not show significant enzymatic activity and induces similar myonecrosis as BthTx-I, when assayed at the same conditions as Barbosa *et al.* [26]. However, an atypical effect presented by BjVIII, and not by other Lys49-PLA2s, is a strong human platelet aggregation activity. We observed that BjVIII induced a dose dependent platelet aggregation, while BthTx-I, PrTx-I and PrTx-II induced a slight and marginal effect. Doses of 1 μg, 3 μg, 9 μg and 12 μg of BjVIII induced, after a time course of 7 minutes, [6 ± 2]%, [19 ± 5]%, [33 ± 4]% and [83 ± 7]% of platelet aggregation (*n* = 6), respectively. On the other hand, doses of 12 μg of PrTx-I, PrTx-II and BthTx-I induced a platelet aggregation of [12 ± 5]%, [8 ± 2]% and [21 ± 6]%, respectively (Fagundes *et al.*, to be published).

**Figure 2.**
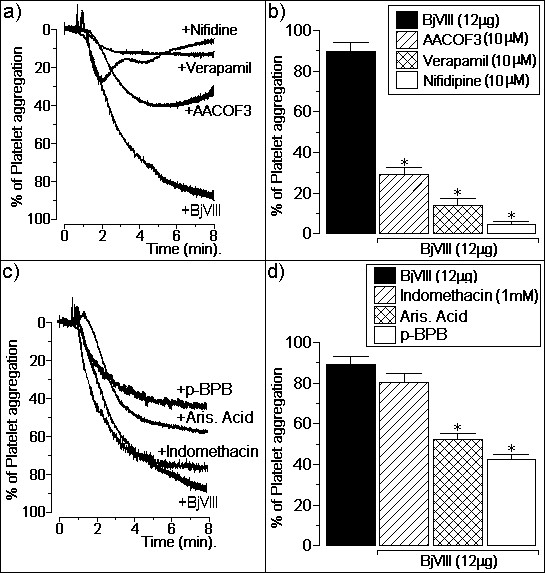
Platelet aggregation activity presented by BjVIII (12 μg). In a) and b), effect of the specific PLA2 inhibitor arachidonyltrifluoromethyl ketone (AACOF3) and calcium ion blockers Nifidipine and Verapamil on platelet aggregation. In c) and d), effect the pre-treatment of BjVIII with Aristolochic acid (Aris Acid), *p*-BPB and Indomethacin.

In platelets previously incubated with 10 μM of arachidonyltrifluoromethyl ketone (AACOF3), a cytosolic phospholipase A2 inhibitor, BjVIII (12 mg) induced a platelet aggregation of [29 ± 4]% (*n* = 6). Under the same experimental conditions, other aliquots of platelets were incubated with 10 μM of verapamil, for 5 minutes, before the PLA2 addition (12 μg), inducing a platelet aggregation of [12 ± 5]% (*n* = 6). The platelet aggregation effect induced by BjVIII was also strongly decreased by the addition of (10 μM) nifidipine: [8 ± 2]% (*n* = 6) (Figures 2a and 2b).

Our results clearly show that specific inhibition with AACOF3 has an important role in platelet aggregation induced by BjVIII. Kramer *et al.* [27] demonstrated that thrombin activates cytosolic PLA2 by promoting an increase in Ca^2+^ influx, which significantly increases the cytosolic calcium concentration. A common event associated to the signal transduction cascade, that occurs during platelet aggregation by thrombin, ADP or collagen, involves phosphorylation of specific proteins, such as mitogen-activated protein and endogenous membrane PLA2 enzyme, which can, in turn, hydrolyze arachidonic acid, forming thromboxane A2, a known activator of platelet aggregation [7, 28].

In addition, three different aliquots of BjVIII, with 12 μg each, were incubated with aristolochic acid sodium salt (Aris Acid), *p*-bromophenacyl bromide (*p*-BPB) and indomethacin. The chemical treatment of BjVIII with Aris Acid and with *p*-BPB induced a platelet aggregation of [52 ± 4]% and [43 ± 4]% (*n* = 6), respectively. Previous incubation with 1 mM indomethacin for 5 minutes did not have any significant effect on the platelet aggregation activity of BjVIII (Figures 2c and 2d).

The treatment of BjVIII with *p*-BPB significantly decreased the platelet aggregation induced by native BjVIII. *p*-BPB is commonly used for alkylation of histidine residues of enzymatically active PLA2s, without modifying other residues present in the polypeptide chain of such enzymes. Alkylation of Lys49-PLA2 myotoxins from *Bothrops pirajai* reduced myotoxicitiy by 40–50% and edema-inducing activity by 15–20%, without significantly changing their ability to disrupt negative liposomes [25, 29]. Aristolochic acid has been characterized as a specific inhibitor of secretory PLA2 and its mode of action, by its ability to enter the substrate binding hydrophobic channel of PLA2 [30]. Treatment of BjVIII toxin with Aris Acid also moderately decreased platelet aggregation induced by this PLA2.

Historically, it was believed that PLA2s exert their pharmacological effects through hydrolysis of cellular phospholipids. However, attempts to correlate these effects with catalytic activity of PLA2s were unsuccessful [31]. Furthermore, there are several catalytically inactive PLA2s which present pharmacological effects [32, 33]. Structure-function studies by chemical modification of amino acids, structural comparison of catalytically and non-catalytically active PLA2s, and use of PLA2 antibodies have suggested the presence in PLA2s of pharmacological domains distinct from the catalytic site [31, 34, 35]. Some years ago, Kini & Evans proposed a model to explain different pharmacological effects of PLA2s [31]. This model was based on the presence of specific binding sites located on the surface of target cells which have high affinities only for toxic PLA2s. Subsequent to this primary binding step, the toxic PLA2s would induce its pharmacological effect by mechanisms either dependent on or independent of phospholipid hydrolysis.

These platelet aggregation activity results point to the existence of molecular regions, distinct from the active site, responsible, at least partially, for pharmacological properties of BjVIII. Crystallographic characterization of BjVIII should provide detailed structural information that is expected to shed light on the paradoxical behavior for this type of PLA2.

### 3.3. Preliminary X-ray diffraction analysis

**Figure 3.**
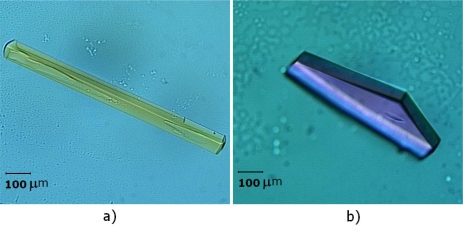
Crystals of BjVIII obtained using the hanging-drop vapour-diffusion method. a) *P*2_1_2_1_2_1_ crystal form and b) *P*3_1_21 crystal form. Images were recorded using polarized light.

Two crystal forms were obtained after refinement of the initial crystallization conditions. A first crystal form grown from a solution containing 0.1 M sodium citrate, pH 8.5, 20% *v/v* 2-propanol and 18% *w/v* PEG 4000, belongs to the orthorhombic space group *P*2_1_2_1_2_1_, with unit-cell parameters *a* = 48.4 Å, *b* = 65.3 Å, *c* = 84.3 Å (Figure 3a).

A second crystal form, belonging to the trigonal space group *P*3_1_21 with cell-dimensions *a* = *b* = 55.7 Å, *c* = 127.9 Å, was obtained from a solution containing 0.2 M ammonium sulfate, 0.1 M MES, pH 8.0 and 28% *w/v* PEG MME 5000 (Figure 3b). Both crystals forms grew within approximately 2 weeks.

**Figure 4.**
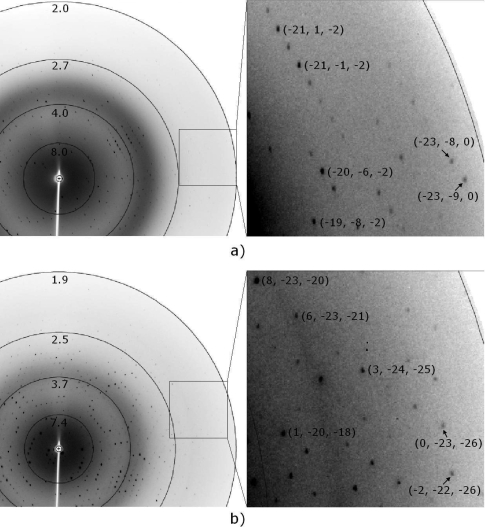
Typical diffraction images with resolutions circles drawn at 2.0 Å, 2.7 Å, 4.0 Å, 8.0 Å resolution for the *P*2_1_2_1_2_1_ space group and 1.9 Å, 2.5 Å, 3.7 Å, 7.4 Å resolution for the *P*3_1_21 space group. a) A 1° oscillation frame from *P*2_1_2_1_2_1_ crystal form and b) a 0.4° oscillation frame from *P*3_1_21 crystal form. A close-up of the outer edge including diffraction spots with their respective indices *hkl* is also shown.

X-ray diffraction data were collected using an oscillation range of 1° and 0.4° to a maximum resolution of 2.0 Å and 1.9 Å for the orthorhombic and trigonal crystals, respectively (Figure 4). Crystal parameters and data-collection statistics are summarized in Table 1. Calculations using the Matthews coefficient [36] suggested the presence of two molecules per ASU for both crystal forms. The primary sequence search and alignments were made using *ENTREZ* and *BLAST* [37]. The complete amino acid sequence of BjVIII comprises 121 amino acid residues (~13.6 kDa; Fagundes *et al.*, to be published).

A *BLAST* search against the PDB database showed that BjVIII has an amino acid sequence identity of 98% with BnSP-7 PLA2 from *Bothrops neuwiedi pauloensis* venom. Therefore, the 2.2 Å resolution BnSP-7 crystal structure (PDB code 1PA0) [38] was used for Molecular Replacement in both crystal forms of BjVIII.

The BnSP-7 crystal structure belongs to the space group *P*3_1_21, with two monomers in the ASU. In the case of the BjVIII crystal belonging to the trigonal space group, a single clear molecular replacement solution was found using the BnSP-7 ASU dimer (chains A and B) as a search model, with a correlation coefficient of 66.3% and *R* factor of 38.8% after fitting. However, for the BjVIII orthorhombic crystal, this procedure was not successful, suggesting a different molecular arrangement in the ASU. For this reason, the phase problem in the space group *P*2_1_2_1_2_1_ was solved in a two step Molecular Replacement procedure, where the two molecules present in the ASU, anticipated by the solvent content analysis, were found using the chain A of the BnSP-7 model as a search model. After fitting, an overall correlation coefficient of 62.4% and an *R* factor of 39.1% were obtained.

**Figure 5.**
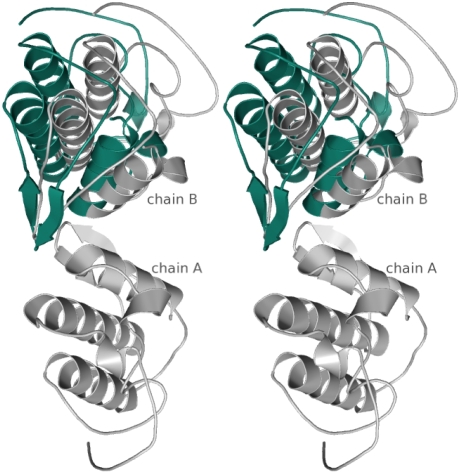
Stereo view of the superposition of the dimers present in the ASU of BjVIII crystals (space groups *P*3_1_21 and *P*2_1_2_1_2_1_). The models shown were built from the search models after application of the molecular replacement solutions, as described in text. The transformation matrix calculated from the superposition of chain A in space group *P*2_1_2_1_2_1_ onto the corresponding chain in space group *P*3_1_21 (fixed) was applied subsequently to the entire dimer present in the ASU of space group *P*2_1_2_1_2_1_. After transformation, chain A coordinates are coincident and, for clarity, for the space group *P*2_1_2_1_2_1_ only the chain B is shown (colored in green). The entire dimer of the space group *P*3_1_21 is shown in gray. The figure unequivocally indicates a difference in the relative orientation of chain B with respect to the chain A. Figures were prepared using *PyMOL* (DeLano Scientific, San Carlos, CA, http://pymol.sourceforge.net) and edited with *GIMP* (http://www.gimp.org) under Linux.

In both crystal forms of BjVIII, a dimer is present in the ASU. In order to investigate structural differences in the molecular arrangement of the ASU contents, a superposition was carried out. A single chain of each dimer (space groups *P*3_1_21 and *P* 2_1_2_1_2_1_) was chosen to calculate the transformation matrix, which was subsequently applied to the entire dimer. The superposition is illustrated in Figure 5.

As expected, the molecules present in the ASU of the orthorhombic space group are arranged in a slightly different manner from those in the space group *P*3_1_21.

## 4. Concluding remarks

A novel non-catalytically active Lys49-PLA2, BjVIII, was purified to a high degree using two chromatographic steps. Electrophoretic analysis indicated that native BjVIII is dimeric in solution, similar to other Lys49-PLA2 homologues. We verified through pharmacological assays that BjVIII presents an atypical effect on human platelet aggregation for the Lys49-PLA2 family. We also have established crystallization conditions for two crystal forms of native BjVIII. The ASU in both space groups, *P*3_1_21 and *P*2_1_2_1_2_1_, can accommodate two molecules, however, in a different arrangement as observed from a comparison of the Molecular Replacement solutions. Complete model building and crystallographic refinement of both structures are currently in progress. The results of this study should provide detailed information about structural features of BjVIII, possibly related to the strong human platelet aggregation activity unusual for this type of PLA2.

## 5. Acknowledgements

This work was supported by Fundação de Amparo à Pesquisa do Estado de São Paulo (FAPESP) and Conselho Nacional de Desenvolvimento Científico e Tecnológico (CNPq). Crystallographic data was collect at LNLS under proposal D03B-MX1-6352. We are also grateful to Prof. Carol Collins (IQ/Unicamp) for carefully reading the manuscript and assistance with language revision.

## Figures and Tables

**Table 1.** Crystal parameters and X-ray data-collection statistics. Values in parentheses refer to the last resolution shell.

Crystal form	Orthorhombic	Trigonal
Wavelength used (Å)	1.425	1.425
Space group	*P*2_1_2_1_2_1_	*P*3_1_21
Unit-cell parameters		
*a* (Å)	48.4	55.7
*b* (Å)	65.3	55.7
*c* (Å)	84.3	127.9
Unit-cell volume (Å^3^)	266666.7	344131.9
V_*M*_ (Å^3^ Da ^−1^)	2.44	2.10
Solvent content (%)	49.69	41.53
ASU contents (molecules)	2	2
Resolution range (Å)	84.2−2.0 (2.1−2.0)	48.3−1.9 (2.0−1.9)
No. of images	197	237
No. of measured reflections	136881 (14411)	103843 (11729)
No. of unique reflections	19116 (2226)	20906 (2525)
Completeness (%)	96.5 (78.6)	97.2 (81.8)
Multiplicity	7.2 (6.5)	5.0 (4.6)
〈*I*/σ(*I*)〉	20.0 (4.6)	22.5 (6.4)
†$R_{\text{merge}}^{†}$ (%)	7.9 (35.4)	4.9 (17.7)

† *R*_*merge*_= Σ_h_ Σ_*l*_ |*I*_h*l*_ − 〈*I*_h_〉|/Σ_h_ Σ*l* 〈*I*_h_〉, where *I*_h*l*_ is the *l*th observation of reflection h and 〈*I*_h_〉 is the weighted average intensity for all observations *l* of reflection h.
